# Organs-on-a-Chip Recapitulating the Gut–Islets Axis for Endocrine Hormone Secretion Regulator Evaluation

**DOI:** 10.34133/research.0923

**Published:** 2025-10-09

**Authors:** Ji Sun, Zhuhao Wu, Jingbo Li, Luoran Shang, Yuanjin Zhao, Ling Li

**Affiliations:** ^1^ Department of Endocrinology, Zhongda Hospital, School of Medicine, Southeast University, Nanjing 210009, China.; ^2^ Department of Rheumatology and Immunology, Institute of Translational Medicine, The Affiliated Drum Tower Hospital of Nanjing University Medical School, Nanjing 210002, China.; ^3^ Zhongshan-Xuhui Hospital and the Shanghai Key Laboratory of Medical Epigenetics the International Co-laboratory of Medical Epigenetics and Metabolism Ministry of Science and Technology, Institutes of Biomedical Sciences, Fudan University, Shanghai 200032, China.

## Abstract

Organ-on-a-chip is emerging as a vital platform for in vitro modeling of biological systems. However, its application in the gut–islets axis and assessing regulators of endocrine hormone secretion has yet to be explored. Here, we developed an organ-on-a-chip platform featuring a microfluidic chip with scaffolds of a closed-packed porous structure to recapitulate the characteristics of the gut–islets axis for bile acid (BA) evaluation. The scaffolds were fabricated by negative replication of assembled droplet templates, enabling intestinal L-cells and pancreatic β-cells to form uniform spheroids. The scaffolds were embedded within a well-designed cascading microfluidic chip capable of generating a concentration gradient. Through this, the assessment of different concentrations of BAs in promoting GLP-1 and insulin secretion was achieved, with results consistent with previous studies, indicating the high accuracy of our platform. This novel system holds promise for evaluating other drugs or signaling molecules involved in glucose homeostasis, offering a new avenue for metabolic drug discovery.

## Introduction

Gut–islets axis is a key endocrine signaling axis that influences islet function by modifying the gut microenvironment and its metabolic activities [1]. Studies have demonstrated that hormones secreted by intestinal L-cells, such as glucagon-like peptide-1 (GLP-1), are essential in modulating the gut–islets axis. GLP-1 stimulates the islets to enhance insulin secretion and lower blood glucose levels [2,3]. This paves the way for the research of endocrine hormone secretion regulators such as bile acids (BAs) and BA analogs for diabetes treatment based on the regulation effect of the gut–islets axis. Beyond stimulating GLP-1 release, BAs also signal via farnesoid X receptor (FXR) and Takeda G protein-coupled receptor 5 (TGR5) [4–6]. Although BAs and BA analogs have been recognized as important signaling molecules with potential benefits for improving glucose regulation and homeostasis [6–9], the interactions within the gut–islets axis are complex, and in vitro models that can recapitulate the characteristics and interplay between gut and islet are unexplored. Therefore, new techniques with the aim to deepen the understanding of the gut–islets axis and improve the evaluation of BAs are highly desired.

**Fig. 1.**
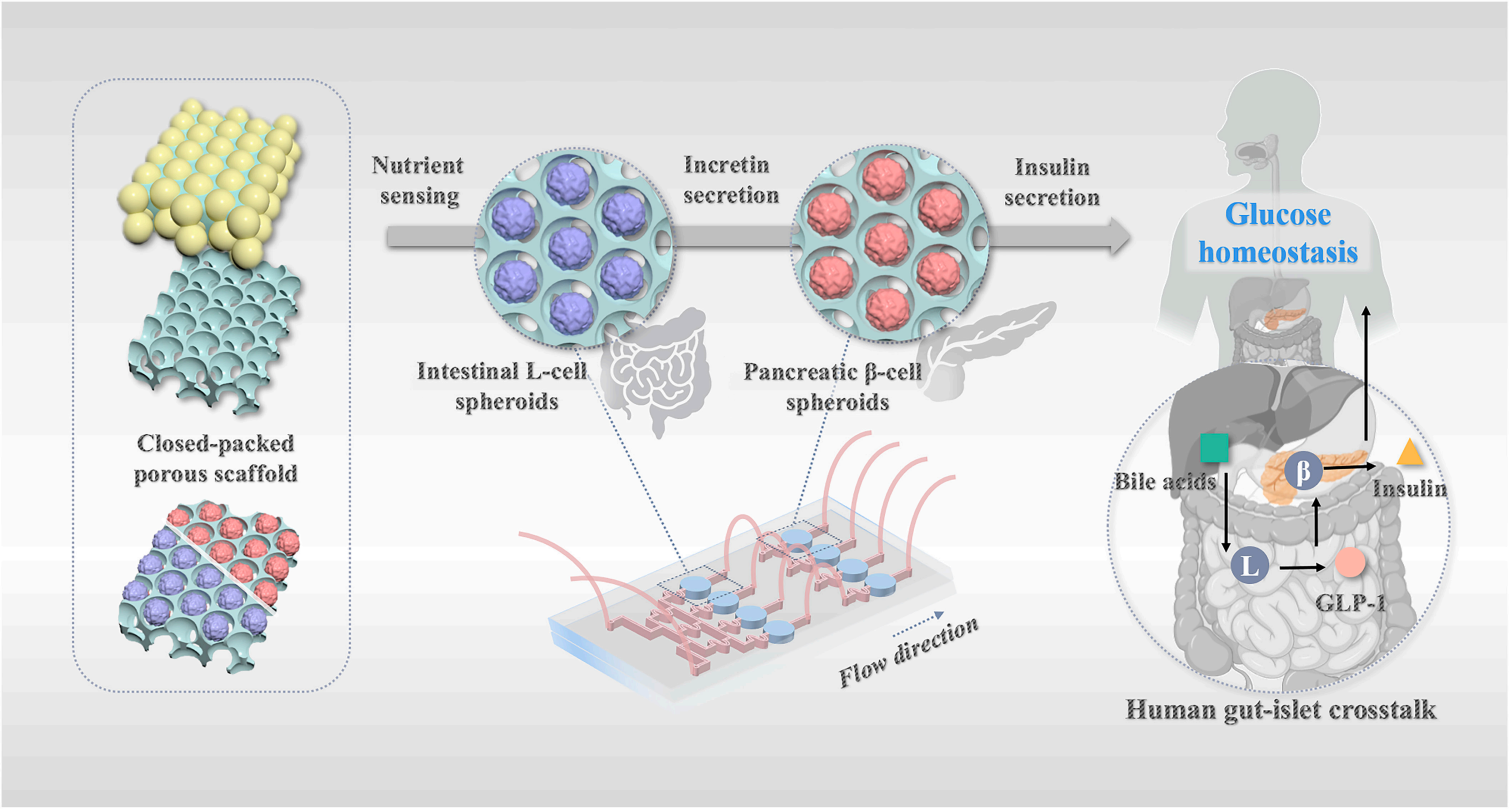
Schematic diagram of the organ-on-a-chip recapitulating the gut–islets axis for BA evaluation. A scaffold is constructed with a closed-packed porous structure that facilitates the formation of β-cell and L-cell spheroids. Such scaffold is integrated within a gradient microfluidic cascading chip. This system enables precise and high-throughput assessment of BAs’ effects on GLP-1 and insulin secretion.

In this study, we develop an organ-on-a-chip platform incorporating a closed-packed porous structured scaffolds for controllable culture of cell spheroids for recapitulating the gut–islets axis and evaluation of BAs, as illustrated in Fig. 1. Organ-on-a-chip is a biomimetic microsystem that integrates the microfluidic technology to mimic the physiological microenvironment for cells, tissues, and organs [10–14]. Organ-on-a-chip aims to replicate the essential functions of specific human tissues and organs, with successful examples including lung [15–17], liver [18,19], pancreatic islet [20,21], and intestine [22,23]. Attractively, the incorporation of biomaterial scaffolds, such as those with an interconnected porous structure, enables 3-dimensional (3D) biomimetic cell culture and the formation of cell spheroids, providing a more physiologically relevant platform for biomedical research [10,24–28]. It is thus conceived that by integrating porous scaffolds with microfluidics, an organ-on-a-chip platform for cell spheroid culture can be established for the simulation of the gut–islets axis.

Herein, we developed an organ-on-a-chip platform with a scaffold with closed-packed porous structure to facilitate the formation of β-cell and L-cell spheroids. Scaffolds featuring adjustable pore sizes and customizable microstructures were fabricated by replicating the close-packed arrangement of monodisperse droplet templates. Due to the uniform and highly ordered porous architecture of the scaffolds, β-cells and L-cells gathered within the pores and eventually developed into uniform β-cell and L-cell spheroids. Additionally, a cascaded microfluidic system was integrated to enable the investigation on glucose-dependent insulin secretion mediated by GLP-1 on the chip. Furthermore, the chip allows for the screening of BAs based on the establishment of a concentration gradient [29,30]. The results demonstrated that hyocholic acid (HCA) exhibited the favorable effect of GLP-1 and insulin secretion, consistent with previous studies. The developed organs-on-a-chip offers not only an innovative platform for investigating the gut–islets axis but also potential applications in metabolic agent discovery and personalized medicine.

## Results and Discussion

**Fig. 2.**
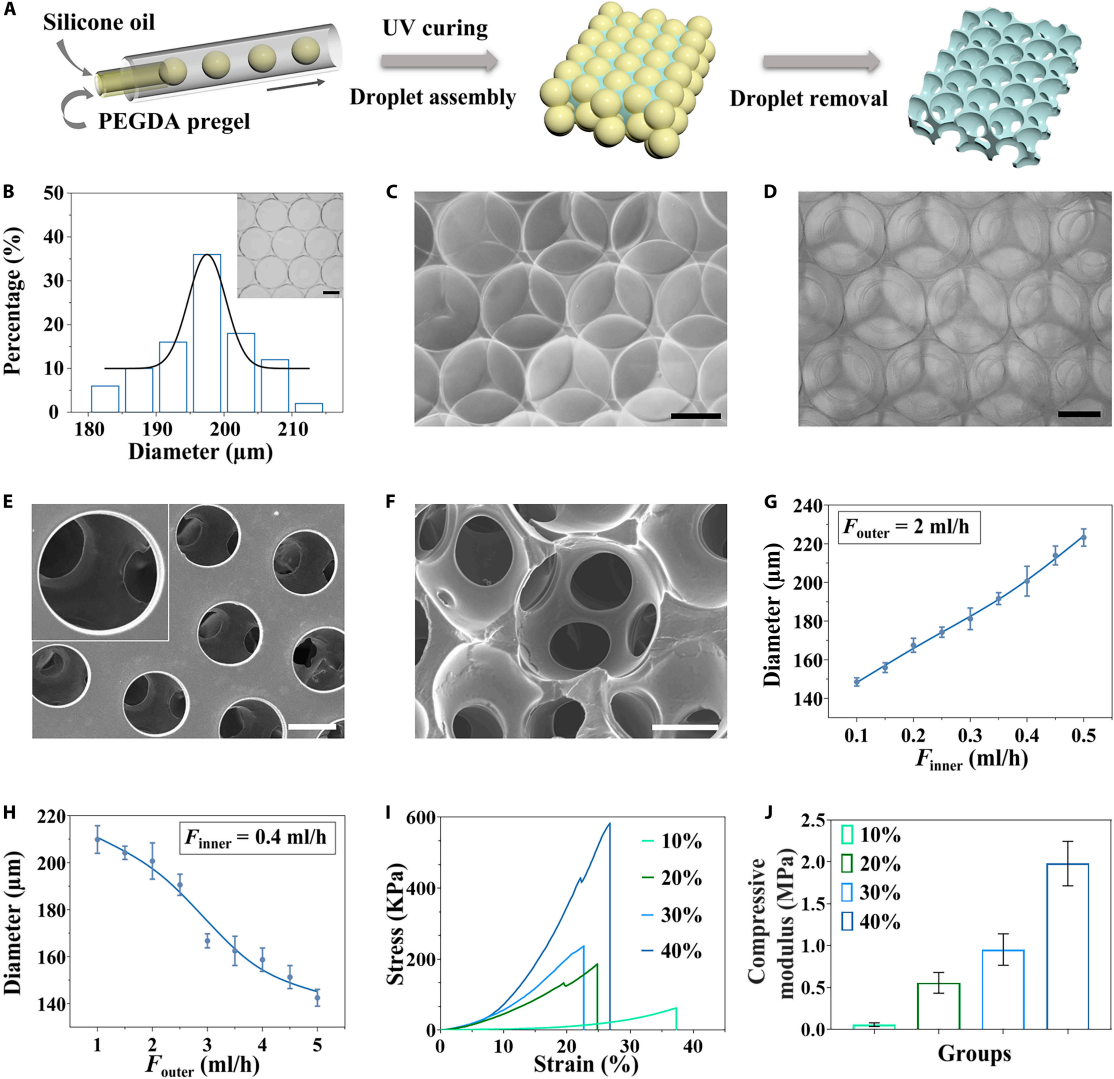
Characterization of the droplet template and the PEGDA scaffold. (A) Schematic illustration of the PEGDA scaffold preparation process. (B) Size distribution of the droplets and the microscopic images of a monolayer droplet assembly (*n* = 50). (C) Representative microscopic image of multilayer droplet assembly. (D) Representative microscopic image of the PEGDA scaffold. (E and F) SEM image of the PEGDA scaffold (top view and section view). Scale bars, 100 μm. (G) Relationship between the diameter of the droplets and the inner flow rate (*n* = 10). (H) Relationship between the diameter of the droplets and the outer flow rate (*n* = 10). (I and J) Stress–strain curves and compressive modulus of different concentrations of PEGDA hydrogels (*n* = 3).

To fabricate the interconnected porous scaffolds, we employed the microfluidic technique to produce oil-in-water (O/W) single-emulsion droplet templates, as illustrated in Fig. 2A. The microfluidic device consisted of cylindrical glass capillaries, with the inner and outer ones arranged coaxially inside a square capillary. The outer phase was polyethylene glycol diacrylate (PEGDA) pregel solution, while the inner phase consisted of methyl silicone oil. At the outlet of the inner phase capillary, O/W droplets were formed under the action of interfacial tension and shear force. The produced droplets showed high sphericity and consistent size (Fig. 2B), facilitating rapid assembly into a close-packed lattice structure (Fig. 2C). The PEGDA pregel was then polymerized under ultraviolet (UV) light to form hydrogel scaffolds, and the interconnected porous scaffold was ultimately created by eliminating the droplet templates. Optical microscopy of the formed PEGDA scaffolds displayed a well-organized and uniform porous structure (Fig. 2D), while scanning electron microscopy (SEM) revealed the interconnections among the voids (Fig. 2E and F).

Utilizing microfluidic techniques for the preparation of droplet templates enables manipulation of both outer and inner phase flow rates, allowing for the regulation of desired droplet diameter and corresponding scaffold pore size [31–33]. We observed that the size of the generated droplets increased with a higher dispersion phase flow rate and decreased when the continuous phase flow rate was elevated (Fig. 2G and H). Hence, by altering the flow rates of the 2 phases, we were able to produce scaffolds with varying pore sizes. Additionally, we explored the shrinkage and mechanical characteristics of PEGDA hydrogels across 4 varying concentrations (10%, 20%, 30%, and 40%, v/v). It was observed that PEGDA hydrogels of different concentrations exhibited varying levels of shrinkage (Fig. S1), and there was an increase in the compressive modulus in correlation with the rising concentration of the hydrogel (Fig. 2I and J). The compressive modulus of the PEGDA scaffolds reflects their intrinsic stiffness, which is a critical determinant for supporting 3D cell culture. Appropriate stiffness provides structural integrity while facilitating the diffusion of oxygen, nutrients, and signaling molecules, thereby enabling stable cell culture under physiologically relevant conditions. Therefore, based on the hydrogel’s shrinkage behavior and mechanical strength, we identified 30% as the optimal PEGDA concentration for further experiments.

Generally, the porous characteristics of the scaffolds would facilitate cell culture and enhance the efficient exchange of oxygen, nutrients, and waste [34,35]. To recapitulate the gut–islet axis on chip, we selected β-cells and intestinal L-cells for spheroid cultivation. β-cells are the principal insulin-secreting cells that regulate glucose homeostasis, whereas L-cells secrete GLP-1, a key incretin that links intestinal nutrient sensing to islet hormone release. The interaction between these 2 cell types embodies the core endocrine communication within the gut–islet axis. To verify the suitability of the scaffolds for 3D cell culture, we initially examined the cytocompatibility of the PEGDA hydrogel. For this purpose, we cultured pancreatic β-cells and intestinal L-cells using Dulbecco’s modified Eagle’s medium (DMEM) supplemented with PEGDA hydrogel extract for a duration of 72 h. Subsequently, both cell types were subjected to Live-Dead double staining analysis. The results revealed that cells in both the hydrogel extract group (the experimental group) and the control group (cells cultured in standard DMEM) sustained high viability rates over the 3-d period (Fig. S2A and B). Additionally, the Cell Counting Kit-8 (CCK-8) assay indicated strong cell proliferation in both groups (Fig. S2C and D). These findings collectively suggested that the PEGDA hydrogel possessed excellent cytocompatibility and supported the growth and proliferation of the subject cell types.

**Fig. 3.**
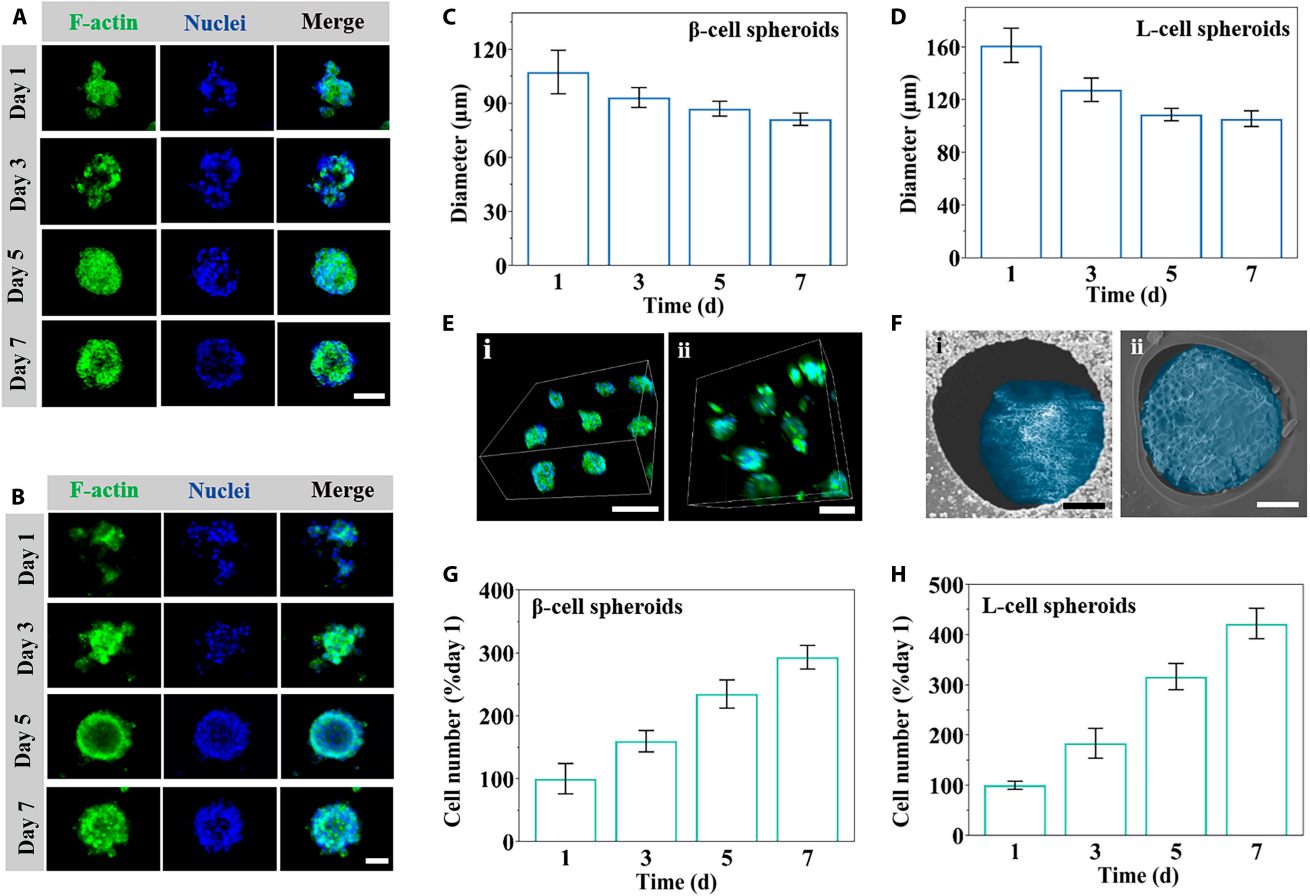
Formation and characterization of the cell spheroids. (A and B) Fluorescence images of (A) β-cell and (B) L-cell spheroids at 1, 3, 5, and 7 d. Scale bars, 50 μm. (C and D) Diameter of (C) β-cell and (D) L-cell spheroids at 1, 3, 5, and 7 d (*n* = 5). (E) 3D reconstructed images of (i) β-cell and (ii) L-cell spheroids at 7th day. Scale bars, 200 μm. (F) SEM image of (i) β-cell and (ii) L-cell spheroids at 7th day. Scale bar, 25 μm. (G and H) Cell proliferation of (G) β-cell and (H) L-cell spheroids at 1, 3, 5, and 7 d (*n* = 3).

To effectively examine cell proliferation and spheroid formation, dense suspensions of β-cells and L-cells were separately seeded into the PEGDA scaffolds with homogeneous cell distribution. Remarkably, the PEGDA scaffolds exhibited a close-packed porous architecture with interconnected channels, which provided efficient oxygen and nutrient transport while maintaining structural stability. The scaffolds demonstrated integrity without notable deformation throughout the cell culture process (Fig. S3). The cell aggregation process was stained with phalloidin and 4′,6-diamidino-2-phenylindole (DAPI). Intensive cell–cell interactions drove the self-assembly and formation of 3D cell spheroids, with increasing incubation times resulting in a reduction of their diameters (Fig. 3C and D). The uniformly porous 3D architecture of the scaffolds was pivotal in ensuring monodispersity and sphericity. 3D cell spheroids ultimately formed after 5 d, and subsequent observations revealed no noticeable alterations in cellular morphology or spheroid diameter (Fig. 3C to E). By 7 d, these cell spheroids were characterized at various depths and specific locations using a confocal laser scanning microscope (CLSM), as shown in Fig. S4. Among them, the β-cell spheroids reached diameters comparable to native pancreatic islets, supporting their physiological relevance [36]. SEM was further employed to examine the surface of these cell spheroids, revealing a dense, smooth surface (Fig. 3F). Moreover, the cell growth persisted as the culture duration increased (Fig. 3G and H). The results demonstrated that the β-cell and L-cell spheroids, cultivated within the PEGDA hydrogel scaffolds, were in a good condition and exhibited sustained proliferation.

**Fig. 4.**
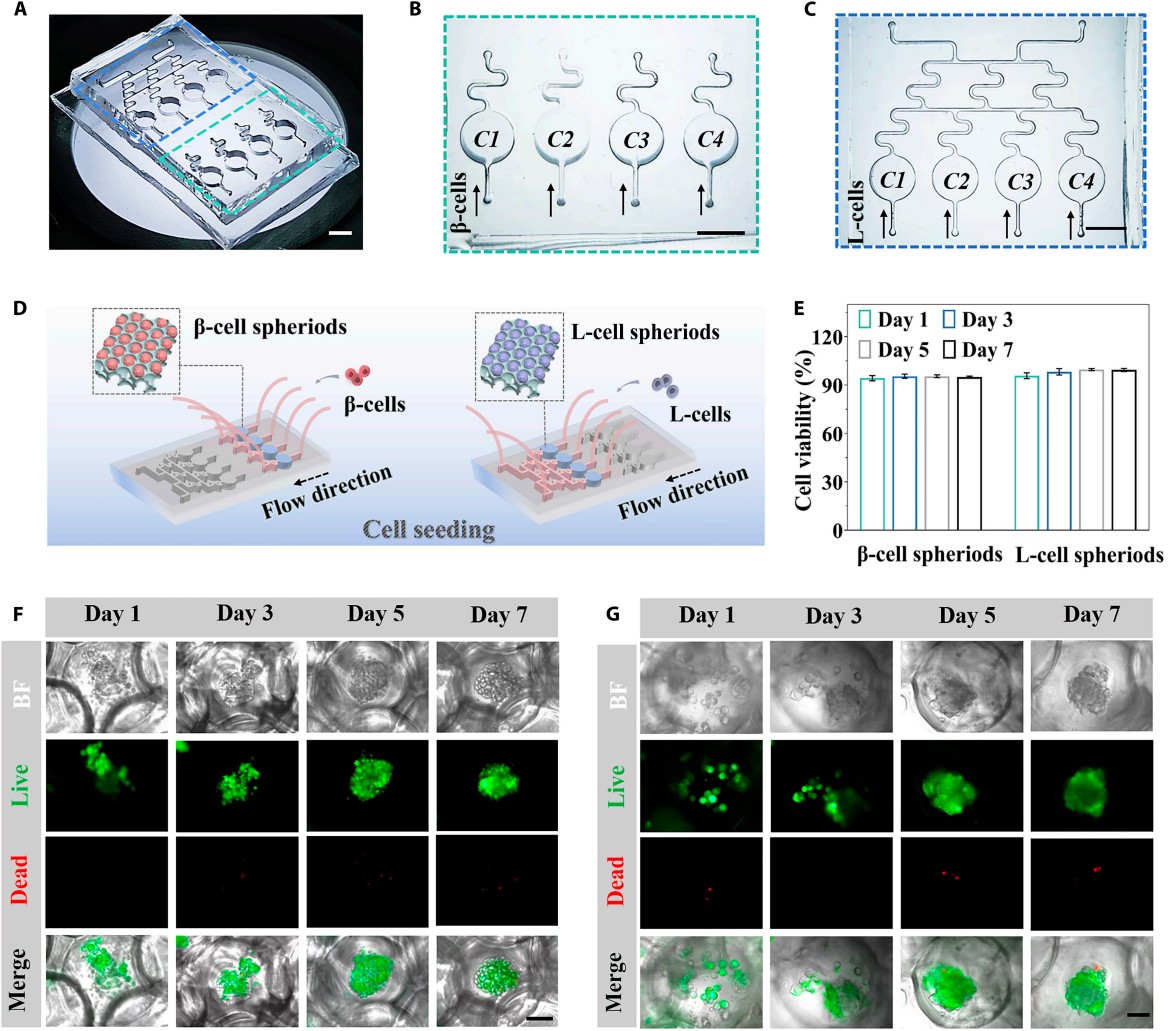
Cell viability of the cell spheroids on the microfluidic chip. (A) Photograph of the chip. (B and C) Images showing the gradient generator and culture chamber of the chip. Scale bars, 5 mm. (D) Schematic diagram of cell seeding in the chip. (E) Cell viability of β-cell spheroids and L-cell spheroids (*n* = 3). (F and G) Fluorescence images of (F) β-cell spheroids and (G) L-cell spheroids were labeled with calcein AM (green) and PI (red) on days 1, 3, 5, and 7. Scale bars, 50 μm.

The formation of stable β-cell and L-cell spheroids laid the foundation for recapitulating the gut–islets axis via organ-on-a-chip. To this end, we constructed a polydimethylsiloxane (PDMS) microfluidic chip consisting of 2 rows of parallel chambers, which were used to embed PEGDA scaffolds for cell spheroid culture. A bifurcated tree-like microchannel on one side was designed, as shown in Fig. 4A to C. This type of channel can act as a concentration gradient generator to rapidly construct multiple mixed gradient solutions, as will be described in subsequent studies on the effects of endocrine hormone regulators [37,38]. Before that, cell spheroid culture on chip was evaluated. Intestinal L-cells and β-cells were injected into corresponding chambers and then seeded in the PEGDA scaffolds within the chambers. After cell depositing, the culture medium was continuously and separately pumped into the distinct chambers designated for β-cells and L-cells through the bifurcated channels by micro-pumps, ensuring a steady supply of nutrients and timely removal of waste products, thereby providing a dynamic microenvironment for cell culture. With this, β-cell and L-cell spheroids were eventually formed (Fig. 4D). Cell viability of mature spheroids was characterized by live-dead staining, and the results were shown in Fig. 4E and F. It was observed that both β-cell and L-cell spheroids preserved a high level of viability throughout the 7-d incubation period.

**Fig. 5.**
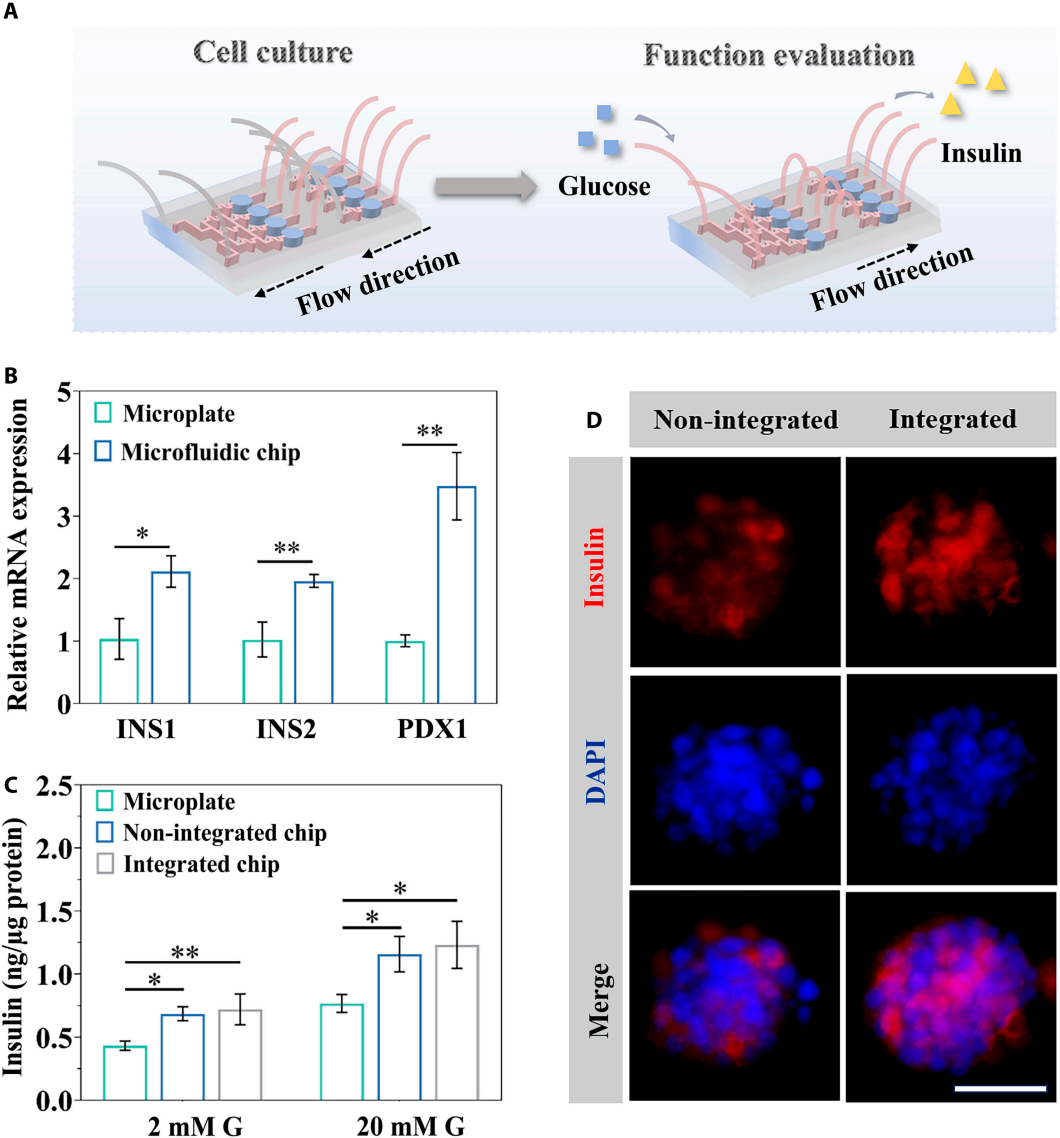
Function evaluation of the simulated gut–islets axis on the microfluidic chip. (A) Schematic diagram of the assembled microfluidic chip, L-cell and β-cell seeding, and function evaluation. (B) Expression of pancreatic function-related genes in β-cells after a 5-d culture on the chip (*n* = 3). *P* value: 0.0105, 0.0056, and 0.0014. (C) β-Cell insulin secretion under varying conditions (*n* = 3). *P* value: 0.0196, 0.0107, 0.0366, and 0.0173. (D) Immunofluorescence image of insulin staining in β-cell spheroids on chip under high-glucose treatment. Scale bar, 50 μm. *P < 0.05, **P < 0.01.

To investigate the function of the β-cell spheroids on chip, we performed reverse transcription quantitative polymerase chain reaction (RT-qPCR) analysis on day 5 to examine the expression of specific mRNAs, including pancreatic and duodenal homeobox 1 (PDX1), INS1, and INS2. PDX1 is crucial for pancreatic development as a transcription factor, and INS1 and INS2 are closely related to the synthesis and secretion of insulin [39–41]. Gene expression analysis showed marked increases in PDX1, INS1, and INS2 in β-cell spheroids cultured in PEGDA scaffolds on the microfluidic chip compared with those cultured in microplates (Fig. 5B). Subsequently, to mimic the glucose-regulatory function of the gut–islets axis in vivo, the outlets of the L-cell spheroid culture chambers were linked with the inlets of the β-cell spheroid culture chambers via microfluidic tubing (Fig. 5A). Here, the non-integrated chip refers to a microfluidic module that supports dynamic culture of a single cell type, whereas the integrated chip denotes the configuration in which the β-cell culture region is interconnected with the L-cell culture region, thereby enabling coupled analysis of their endocrine communication. This integrated system allowed us to observe the impacts of glucose and GLP-1 on insulin secretion. Specifically, glucose not only stimulated insulin secretion but also influenced GLP-1 secretion from the L-cell spheroids, which subsequently affected insulin secretion again [2,42]. Glucose-stimulated insulin secretion was assessed with 2 and 20 mM glucose concentrations in both traditional 2D cell cultures and the spheroids on chip. The results showed that, relative to β-cells cultured in microplates, β-cell spheroids on chip were more effective at secreting insulin in response to glucose, displaying higher amounts of insulin release (Fig. 5C). These findings implied that the glucose solution flow provided continuous stimulation to the β-cell spheroids and the 3D culture system better simulated the in vivo environment of pancreatic islets. As shown in Fig. 5D, insulin immunofluorescence staining was carried out on β-cell spheroids cultured in the microfluidic chip under high-glucose conditions. The developed organ-on-a-chip closely mimics the physiological environment, allowing for a more accurate replication of the gut–islets axis and evaluation of glucose metabolism. This is achieved by integrating L-cell and β-cell spheroids through microfluidic connections, which permits real-time signaling of GLP-1 and insulin in response to glucose stimulation. Unlike traditional 2D culture, the porous PEGDA scaffolds support 3D spheroid growth, maintain cellular integrity, and provide efficient exchange of oxygen and nutrients, thereby preserving endocrine function. The microfluidic flow further simulates physiological circulation, ensuring continuous exposure of spheroids to glucose and secreted hormones. Together, these features enable the system to reproduce core aspects of gut–islet axis regulation in vitro and offer a physiologically relevant platform for studying glucose metabolism.

**Fig. 6.**
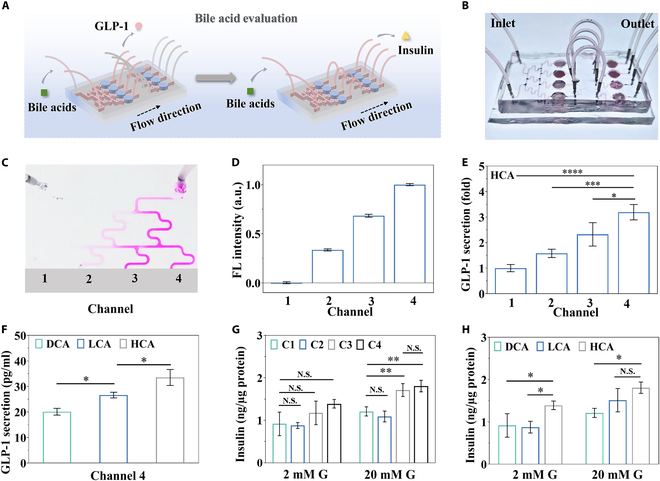
Evaluation results of BAs on organ-on-a-chip. (A) Schematic diagram of BA evaluation. (B) Photograph of the assembled microfluidic chip. (C) Optical microscopic image showing rhodamine B distribution in the microchannels of the microfluidic chip (100 μM rhodamine solution was pumped through the right inlet at 0.1 μl/min flow rate, and phosphate-buffered saline solution was pumped through the left inlet at the same flow rate). (D) Fluorescent intensity of rhodamine B measured at the terminal branches of microfluidic channels 1 to 4 (*n* = 3). (E) GLP-1 secretion of L-cell spheroids incubated in gradient concentration of HCA on chip (*n* = 3). *P* value: <0.0001, 0.0004, and 0.0173. (F) GLP-1 secretion of L-cell spheroids incubated in the concentration of BAs in channel 4 (*n* = 3). *P* value: 0.0201 and 0.0157. (G) Insulin secretion of β-cell spheroids on the integrated microfluidic chips (*n* = 3). *P* value: 0.0069 and 0.0024. (H) Insulin secretion of β-cell spheroids in channel 4 (*n* = 3). *P* value: 0.0498, 0.0356, and 0.0192. *P < 0.05, **P < 0.01, ***P < 0.001, ****P < 0.0001.

To demonstrate the value of the organ-on-a-chip mimicking the gut–islets axis for evaluating regulators of endocrine hormone secretion, the Christmas tree-shaped microchannels were employed to create a concentration gradient (Fig. 6A and B). We conducted numerical simulations on the concentration gradient formed in the branched channel, defining the concentration of a simulated BA molecule at the left inlet as 0 and the right inlet as 1, resulting in a well-established concentration gradient (Fig. S5). Rhodamine B served as a model molecule to visualize this gradient within microfluidic chip (Fig. 6C and D). Fluorescence intensity differed markedly among the terminal branches, aligning with the simulation results and confirming the successful creation of a chemical gradient on the microfluidic chip.

To examine the impact of BAs on GLP-1 secretion from L-cell spheroids, we tested HCA, which has been reported to activate TGR5 and antagonize intestinal FXR, together with lithocholic acid (LCA) and deoxycholic acid (DCA), 2 typical TGR5 agonists. Hanks’ balanced salt solution (HBSS) with or without 50 μM BA was added to 2 separate inlets at a constant flow. The GLP-1 secretion was measured at the outlet of the L-cell spheroid culture chamber on the non-integrated chip. After exposure to BA solutions for 1 h, GLP-1 levels increased in a concentration-dependent manner (Fig. 6E and Fig. S6). Among the 3 agonists, HCA significantly enhanced GLP-1 secretion and resulted in higher amounts of GLP-1 at high concentrations compared to LCA and DCA (Fig. 6F). We further evaluated the impact of different concentrations of BAs on up-regulating GLP-1 and subsequently on stimulating insulin secretion on the integrated chip. Low-glucose (2 mM) and high-glucose (20 mM) Hepes-balanced Krebs–Ringer bicarbonate buffer (KRBH) with or without 50 μM HCA, LCA, or DCA was sequentially added to 2 separate inlets, and insulin secretion was measured at the outlet. The results showed that under high-glucose stimulation, GLP-1 secretion and subsequent insulin secretion were up-regulated in a concentration-dependent manner with increasing HCA concentration. Insulin secretion was significantly elevated in channels 3 and 4 relative to channel 1, although no significant difference was observed between channels 3 and 4 (Fig. 6G). Further, the effects of LCA and DCA were analyzed using the integrated microfluidic chip. It was found that HCA at the concentration of channel 4 significantly enhanced basic insulin secretion on the chip and significantly promoted insulin secretion under high-glucose stimulation compared to DCA (Fig. 6H). Our findings indicate that HCA strongly stimulates GLP-1 secretion and thereby promotes insulin secretion, highlighting its important role in regulating the gut–islet axis.

## Conclusion

Taken together, we have developed an organ-on-a-chip platform with closed-packed porous scaffolds to replicate the gut–islets axis and dynamically evaluate the effects of endocrine hormone regulators. The scaffolds, characterized by organized, uniformly porous structures, were fabricated by negatively replicating water-in-oil single-emulsion droplet assembly template generated by microfluidics. The adjustable pore size and superior connectivity of scaffolds promoted high cellular activity and the formation of β-cell spheroids and L-cell spheroids. Furthermore, by integrating these scaffolds with a microfluidic chip equipped with parallel chambers and microchannels, we have established a dynamic system that supports the sustained and effective culture of cellular spheroids. The integrated microfluidic system allowed for detailed assessment of the functions of β-cell spheroids and L-cell spheroids under various conditions, including glucose-stimulated insulin and GLP-1 secretion, as well as the impact of elevated GLP-1 on insulin secretion. The results underscored the platform’s capability to effectively simulate the physiological conditions and interactions of the gut–islets axis, providing insights into the dynamics of endocrine hormone secretion and the potential effects of substances like BAs on these processes. These findings not only demonstrated the platform’s potential in replicating biological functions and interactions but also established its applicability in dynamically assessing drug responses, thereby offering a valuable tool for future strategies in the development of BA-based metabolic agents and combination of BAs with other glucose-lowering drugs.

## Materials and Methods

### Materials

PEGDA, 2-hydroxy-2-methylpropiophenone (HMPP), sodium dodecyl sulfate (SDS), and poly (ethylene glycol)-block-poly (propylene glycol)-block-poly (ethylene glycol) (F108) were purchased from Sigma-Aldrich, USA. Methyl silicone oil (50 CS) was sourced from Shin-Etsu Chemical Co. Ltd., Japan. The β-cells (MIN6) (RRID:CVCL_0431) and L-cells (STC-1) (RRID:CVCL_J405) were obtained from the American Type Culture Collection (ATCC). Cells were maintained according to ATCC’s recommended protocols. All cell lines were routinely tested and verified to be mycoplasma-free. Live/dead cell double staining kit, CCK-8, and phalloidin were obtained from Beyotime Biotechnology, China. All reagents were used according to the instructions.

### Preparation of the porous PEGDA scaffold

The porous PEGDA scaffold was fabricated by removing self-assembled emulsion template, which was obtained through a single-emulsion droplet microfluidic device. The microfluidic device was assembled by fixing 3 glass capillaries onto a glass slide. The inner phase tube was treated with octadecyltrichlorosilane (OTS) to maintain hydrophobicity. Then, the inner and outer phase tubes were used to deliver the dispersed and continuous phase liquids, respectively. The continuous pregel phase contained PEGDA hydrogel (30%, v/v) and HMPP (1%, v/v). In addition, to stabilize the droplets, 2 surfactants were added (F108 and SDS, 2%, w/v). These solutions were injected into the corresponding tubes through syringe pumps (Longer LSP01-2A, China), and O/W droplets were formed. Following assembly of the droplets into a hexagonal close-packed structure, the pregel solution was cured by UV light. Finally, the oil droplets were removed with n-hexane to generate the porous scaffolds.

### Mechanical property tests of PEGDA hydrogels

The hydrogel was molded into a cylinder measuring 10 mm in height and 10 mm in diameter. Mechanical responses under compression were measured using an Instron-5943 mechanical testing machine at a constant loading speed of 1 mm/min. The stress–strain curves were recorded, and the compressive modulus was determined from the initial linear region of the curves.

### Evaluation of PEGDA hydrogel’s biocompatibility

The biocompatibility of the PEGDA hydrogel was assessed using β-cells (MIN6) and L-cells (STC-1). The viability of cells incubated with PEGDA hydrogel extract was evaluated through calcein AM/propidium iodide (PI) staining. Briefly, both cell lines were initially cultured overnight in a 96-well plate (Thermo, USA). The leachate was then applied to the experimental group, followed by a 72-h incubation. Cell viability was evaluated at 24, 48, and 72 h using calcein AM/PI staining. The PEGDA hydrogel’s cytotoxicity was assessed with a CCK-8 assay by adding 10 μl of CCK-8 solution to each well, followed by a 1-h incubation at 37 °C, and measuring the absorbance at 450 nm using a microplate reader (SYNERGY HTX).

### Formation of the cell spheroids

Prior to cell seeding, the PEGDA scaffolds were washed with 75% ethanol and subsequently rinsed with sterile deionized water. The scaffolds were then sterilized using UV irradiation for 2 h. A cell suspension at 3 × 10^4^ cells/ml was seeded onto the sterilized PEGDA scaffolds. Fresh medium was replenished daily.

### Preparation of the organs-on-a-chip

The microfluidic chip was fabricated by replicating a custom polymethyl methacrylate (PMMA) mold with PDMS. A mixture of PDMS base and curing agent (10:1) was thoroughly blended, degassed under vacuum, and cast into the mold. After curing, the PDMS replica was peeled off to obtain the designed structure. PEGDA scaffolds were then placed into the designated chambers. The patterned PDMS layer was bonded to a flat PDMS substrate using plasma treatment to seal the device. Finally, the assembled chip was sterilized by UV irradiation before cell culture experiments.

### Quantitative real-time PCR analysis

Quantitative real-time PCR (qPCR) was performed using Taq Pro Universal SYBR qPCR Master Mix and specific primers. Glyceraldehyde-3-phosphate dehydrogenase (GAPDH) served as the internal control, and relative transcript levels were determined via the 2^−ΔΔCt^ calculation method.

### GLP-1 measurement

The L-cell spheroids were exposed to varying concentrations of different BAs for 1 h, followed by supernatant collection. Active GLP-1 levels were measured using an active GLP-1 assay kit.

### Glucose-stimulated insulin secretion assay

To assess the biological function of β-cell spheroids in the assembly chips, insulin secretion in response to glucose stimulation was measured on the 5th day. Chips were preincubated in KRBH buffer at 37 °C for 1 h and were incubated for 1 h in 2 and 20 mM glucose. Then, the supernatant was analyzed using a mouse insulin enzyme-linked immunosorbent assay (ELISA) kit.

### Characterizations

The droplet template and the PEGDA scaffold were captured using a stereomicroscope (Olympus SZX16, Japan). The structures of the PEGDA scaffold and cell spheroids were analyzed using field emission SEM (Hitachi SU8010, Japan). Fluorescence images of cell spheroids were captured using a confocal laser scanning microscope (Nikon A1, Japan).

### Statistical analysis

All experiments were conducted in triplicate, and data are presented as mean ± standard deviation. Comparisons between 2 groups was conducted using unpaired Student’s *t* tests, while differences among multiple groups was determined using one-way analysis of variance (ANOVA) with Tukey’s post hoc tests, and differences were considered statistically significant at *P* < 0.05.

## Data Availability

The data that support the findings of this study are available from the corresponding author upon reasonable request.
