# Supplementary material for: Organs-on-a-Chip Recapitulating the Gut–Islets Axis for Endocrine Hormone Secretion Regulator Evaluation
Source: Research (Wash D C). 2025 Oct 9;8:0923. doi: 10.34133/research.0923 (PMC12508524; doi:10.34133/research.0923)
Supplement: Supplementary 1 — Figs. S1 to S6 [file research.0923.f1.docx]

**Supplementary Materials**

**Organs-on-a-chip recapitulating the gut-islets axis for endocrine hormones secretion regulators evaluation**

*Ji Sun^1,2^, Zhuhao Wu^2^, Jingbo Li^1,2^, Luoran Shang^3,^ *, Yuanjin Zhao^2,^ *, Ling Li^1,^ **

Dr. Ji Sun, Jingbo Li, Prof. Ling Li

1. Department of Endocrinology, Zhongda Hospital, School of Medicine, Southeast University, Nanjing 210009, China

E-mail: [lingli@seu.edu.cn](mailto:lingli@seu.edu.cn)

Prof. Luoran Shang

1. Zhongshan-Xuhui Hospital and the Shanghai Key Laboratory of Medical Epigenetics the International Co-laboratory of Medical Epigenetics and Metabolism Ministry of Science and Technology, Institutes of Biomedical Sciences, Fudan University, Shanghai 200032, China
2. mail: [luoranshang@fudan.edu.cn](mailto:luoranshang@fudan.edu.cn)

Dr. Ji Sun, Zhuhao Wu, Jingbo Li, Prof. Yuanjin Zhao

1. Department of Rheumatology and Immunology, Institute of Translational Medicine, The Affiliated Drum Tower Hospital of Nanjing University Medical School, Nanjing, 210002, China

E-mail: yjzhao@njglyy.com

*
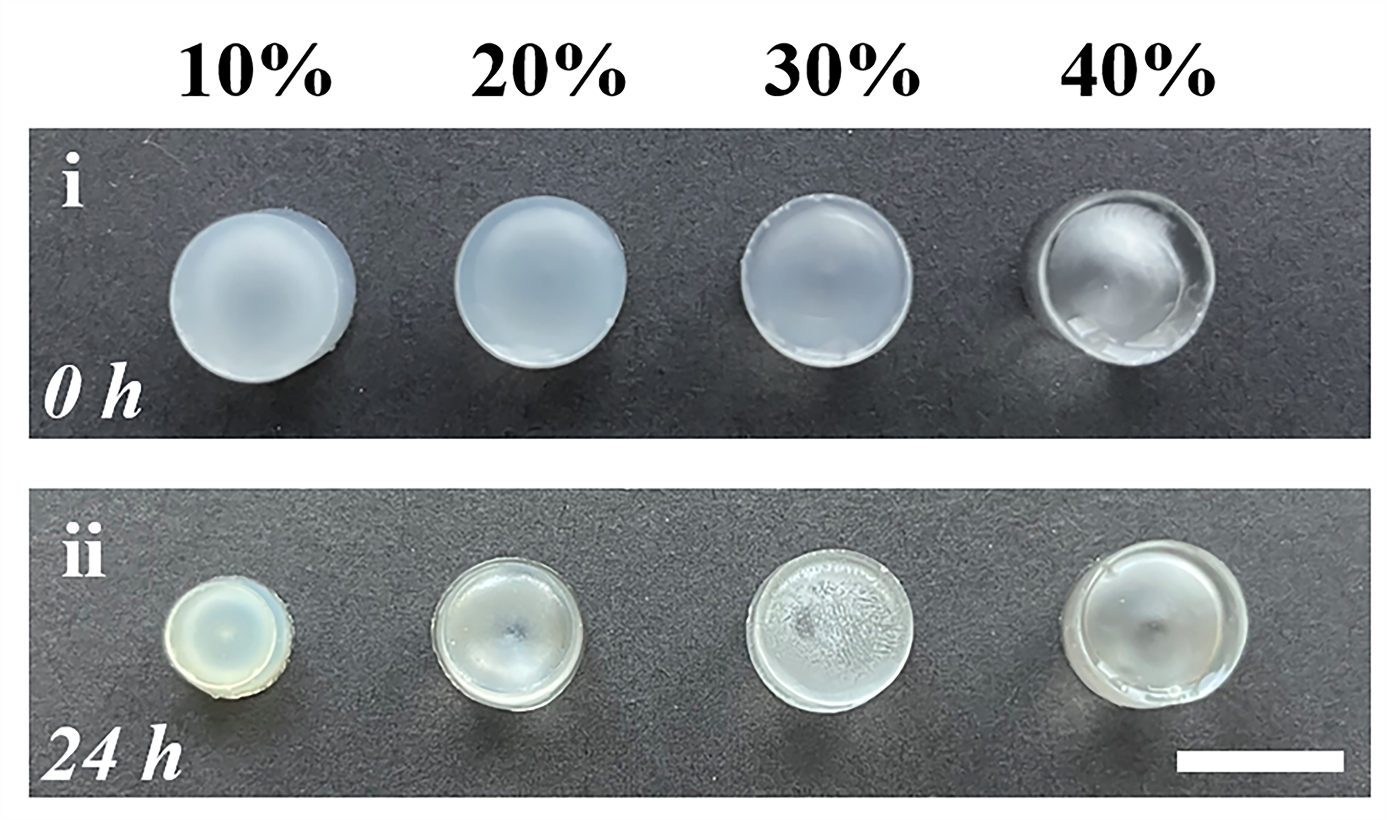
*

**Figure S1.** Photographs of bulk PEGDA hydrogels with different concentrations at 0 h and 24 h. Scale bar is 1 cm.


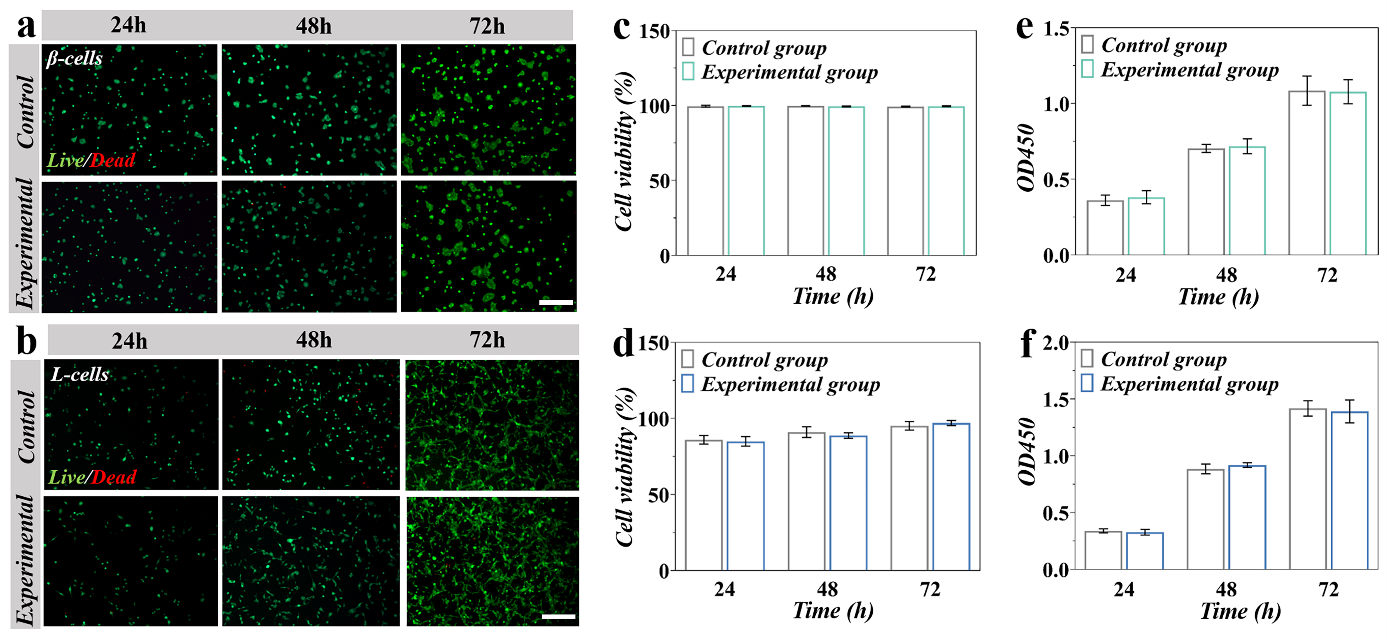


**Figure S2**. Biocompatibility of PEGDA hydrogel. a, b) Live/dead staining of a) β-cells and b) L-cells in the control and experimental groups at 24, 48 and 72h. Scale bars are 100 μm. c, d) Cell viability of c) β-cells and d) L-cells in the control and experimental groups at 24, 48 and 72h. e, f) Cell proliferation of e) β-cells and f) L-cells in the control and experimental groups at 24, 48 and 72h (n=3).

**
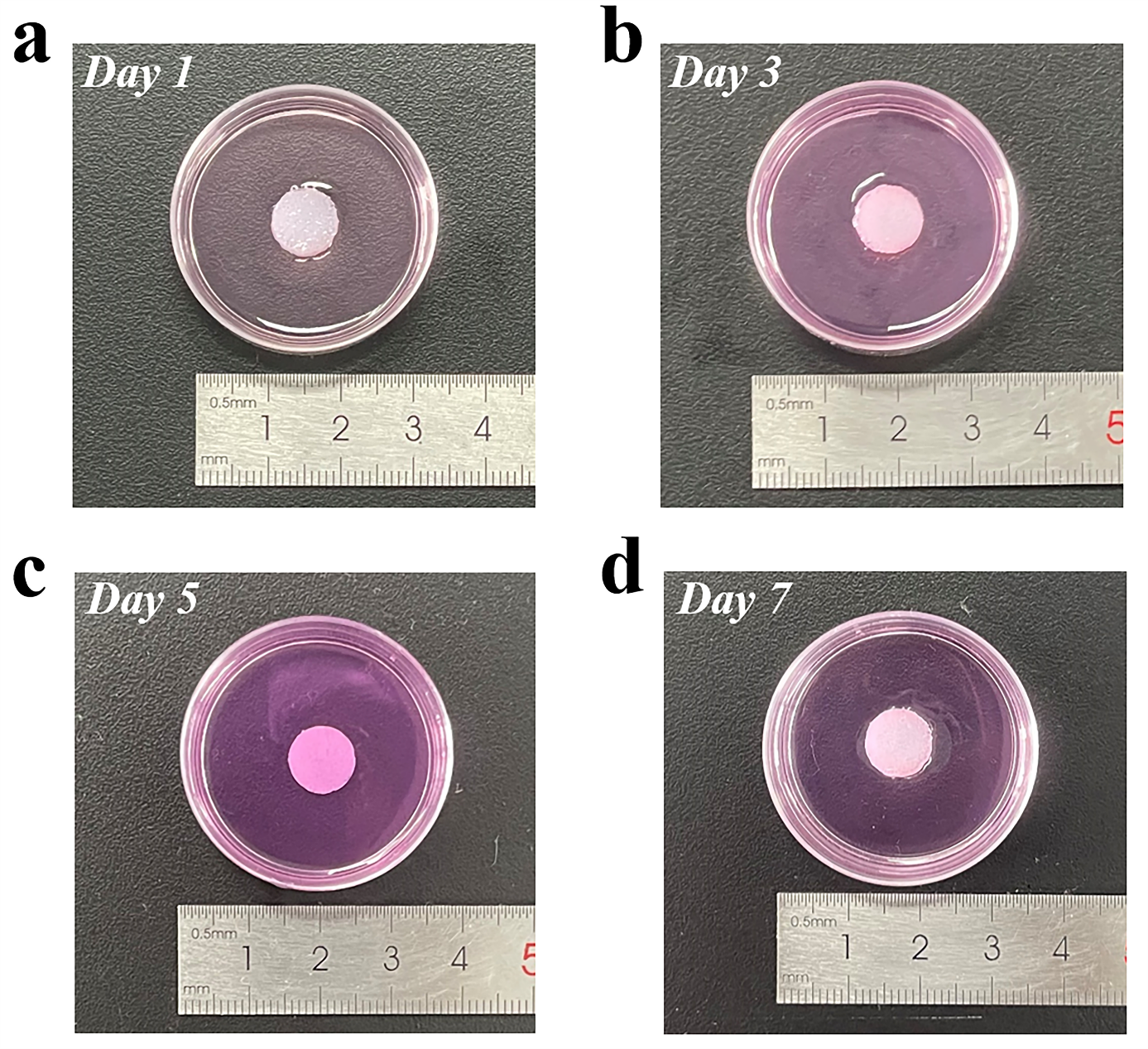
**

**Figure S3.** Images of the PEGDA scaffold during 7-day cell culture.


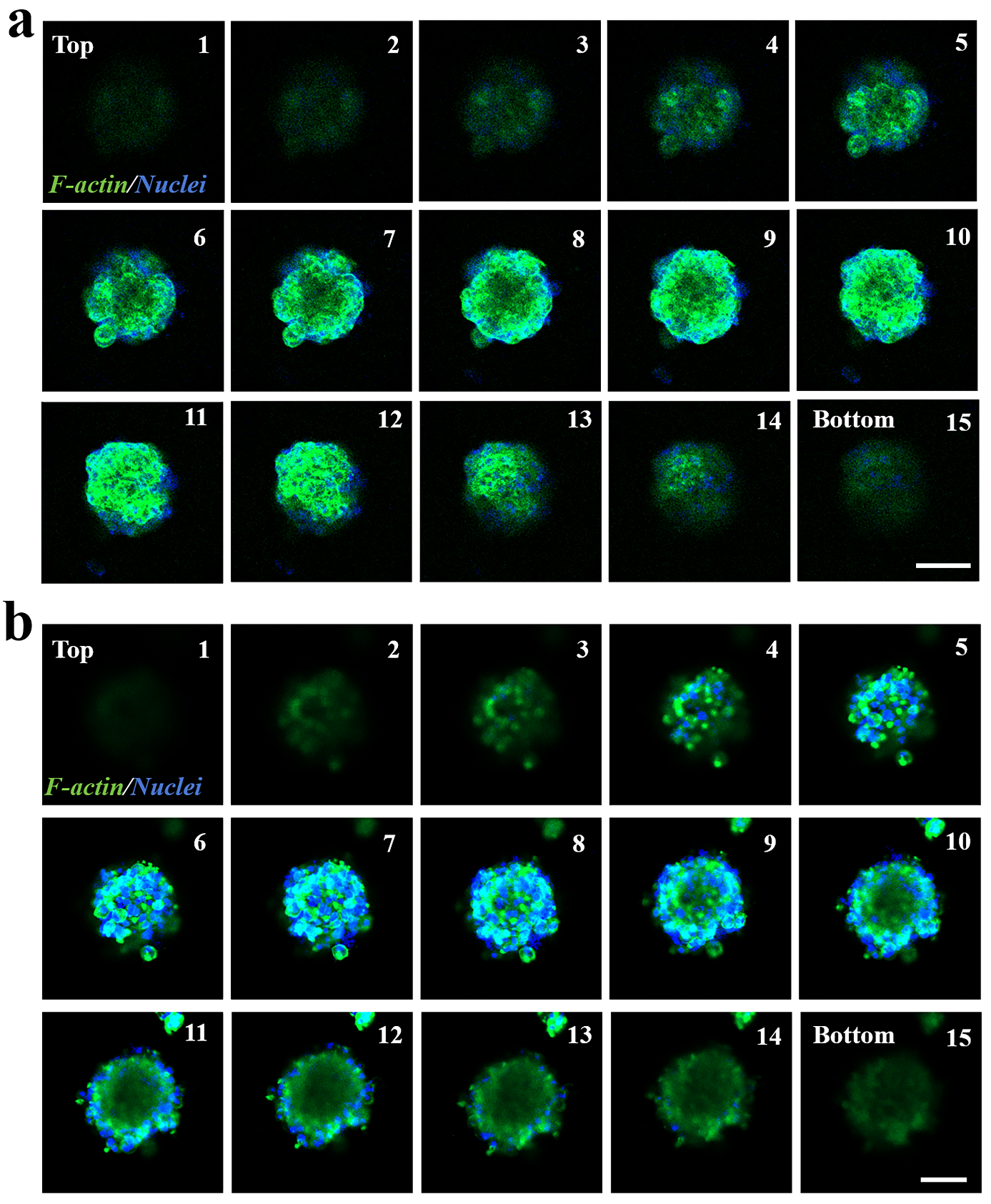


**Figure S4.** The CLSM 3D layer scanning images of a) β-cell spheroids and b) L-cell spheroids. The scale bar is 50 μm.

**
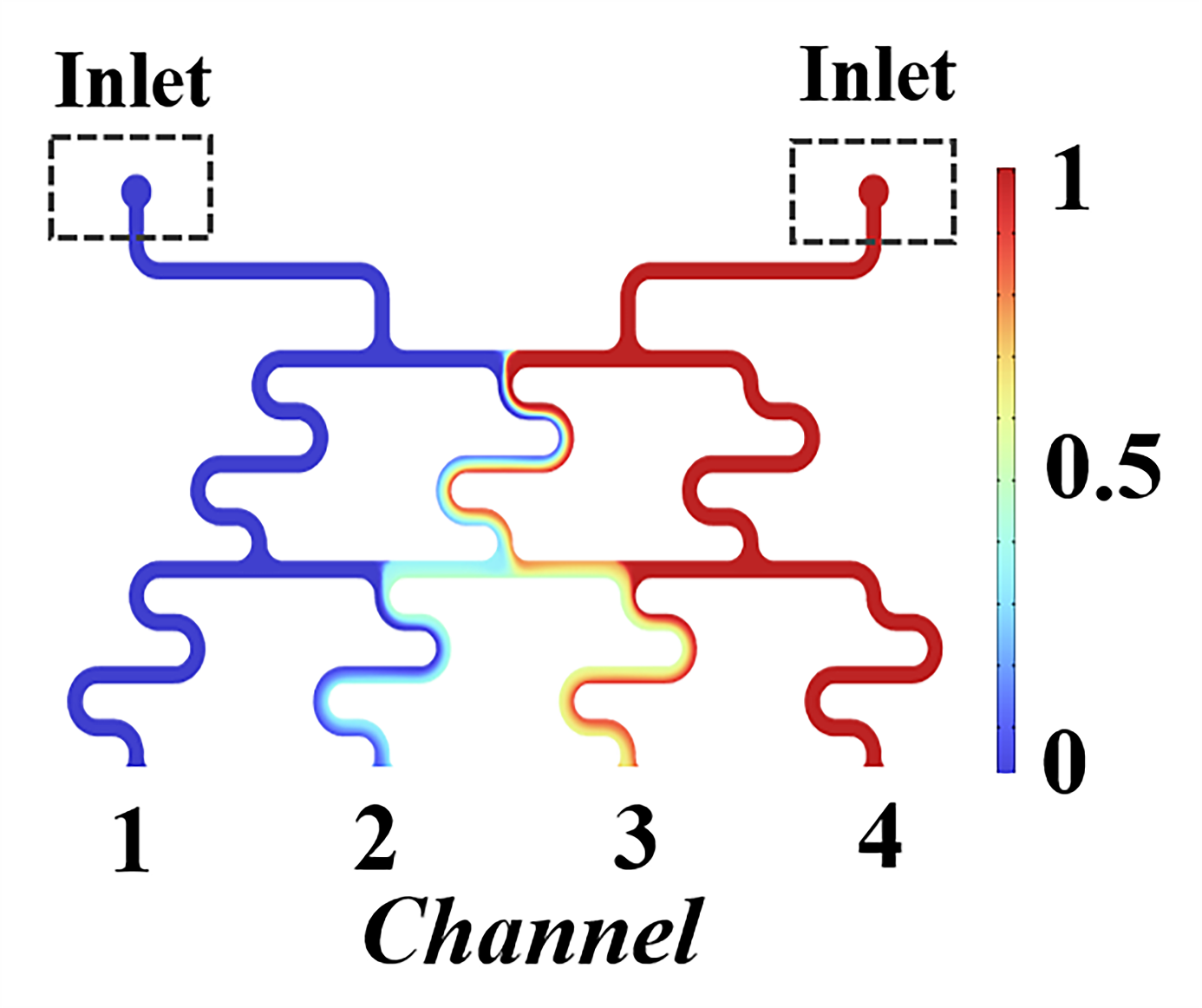
**

**Figure S5.**  Numerical modeling of the concentration distribution inside the microfluidic gradient generator.


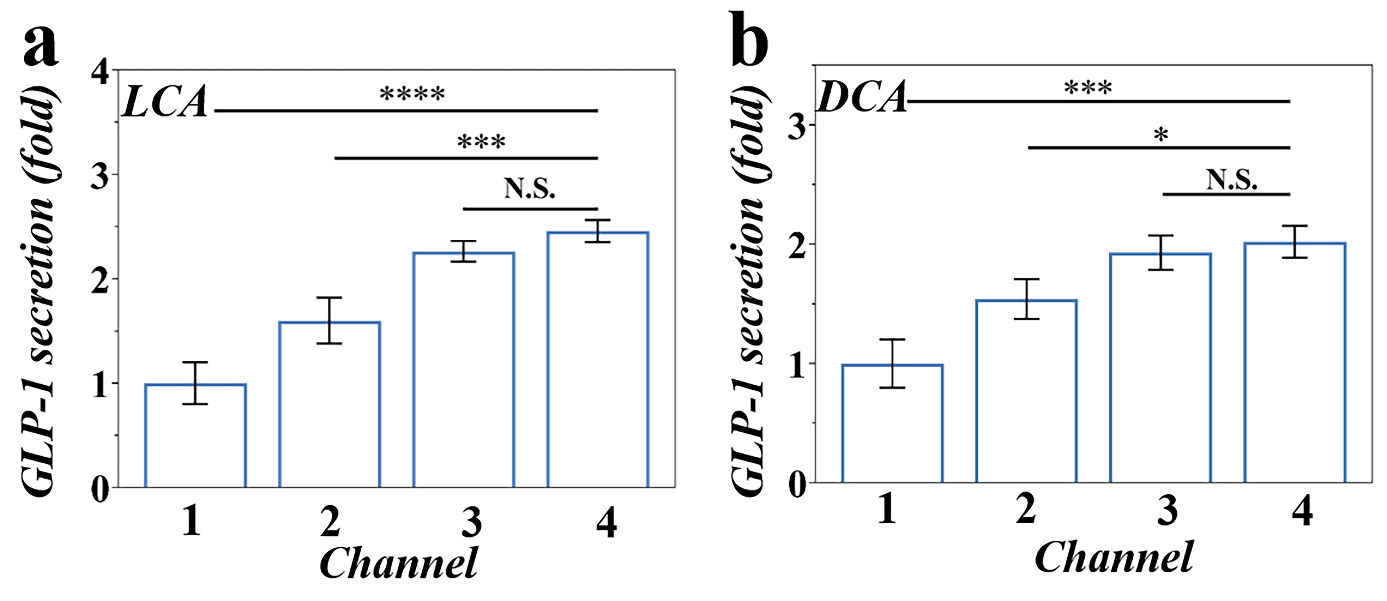


**Figure S6.** GLP-1 secretion of L-cell spheroids incubated in gradient concentration of a) LCA and b) DCA on microfluidic chips (n=3). *P* value for a): <0.0001, 0.001. *P* value for b): 0.0002, 0.0176. *P < 0.05, **P < 0.01, ***P < 0.001, ****P < 0.0001.
